# Pediatric neuropathology practice in a low- and middle-income country: capacity building through institutional twinning

**DOI:** 10.3389/fonc.2024.1328374

**Published:** 2024-05-03

**Authors:** Ahmed Gilani, Naureen Mushtaq, Muhammad Shakir, Ahmed Altaf, Zainab Siddiq, Eric Bouffet, Uri Tabori, Cynthia Hawkins, Khurram Minhas

**Affiliations:** ^1^ Department of Pathology, University of Colorado Anschutz Medical Campus, Aurora, CO, United States; ^2^ Department of Medicine, The Aga Khan University, Karachi, Pakistan; ^3^ Department of Oncology, The Aga Khan University Hospital, Karachi, Pakistan; ^4^ Department of Surgery, The Aga Khan University Hospital, Karachi, Pakistan; ^5^ Neurooncology Program, Division of Hematology/Oncology, The Hospital for Sick Children, Toronto, ON, Canada; ^6^ Global Pediatric Medicine Department, St. Jude Children’s Research Hospital, Memphis, TN, United States; ^7^ Department of Pathology, The Hospital for Sick Children, Toronto, ON, Canada; ^8^ Department of Pathology and Laboratory Medicine, The Aga Khan University Hospital, Karachi, Pakistan

**Keywords:** Low- and lower-middle-income countries, diagnosis, brain tumor, pathology, precision medicine, precision diagnostics, next generation (deep) sequencing (NGS), targeted therapy

## Abstract

**Background:**

Accurate and precise diagnosis is central to treating central nervous system (CNS) tumors, yet tissue diagnosis is often a neglected focus in low- and middle-income countries (LMICs). Since 2016, the WHO classification of CNS tumors has increasingly incorporated molecular biomarkers into the diagnosis of CNS tumors. While this shift to precision diagnostics promises a high degree of diagnostic accuracy and prognostic precision, it has also resulted in increasing divergence in diagnostic and management practices between LMICs and high-income countries (HICs). Pathologists and laboratory professionals in LMICs lack the proper training and tools to join the molecular diagnostic revolution. We describe the impact of a 7-year long twinning program between Canada and Pakistan on pathology services.

**Methods:**

During the study period, 141 challenging cases of pediatric CNS tumors initially diagnosed at Aga Khan University Hospital (AKUH), Karachi, were sent to the Hospital for Sick Children in Toronto, Canada (SickKids), for a second opinion. Each case received histologic review and often immunohistochemical staining and relevant molecular testing. A monthly multidisciplinary online tumor board (MDTB) was conducted to discuss the results with pathologists from both institutions in attendance.

**Results:**

Diagnostic discordance was seen in 30 cases. Expert review provided subclassification for 53 cases most notably for diffuse gliomas and medulloblastoma. Poorly differentiated tumors benefited the most from second review, mainly because of the resolving power of specialized immunohistochemical stains, NanoString, and targeted gene panel next-generation sequencing. Collaboration with expert neuropathologists led to validation of over half a dozen immunostains at AKUH facilitating diagnosis of CNS tumors.

**Conclusions:**

LMIC-HIC Institutional twinning provides much-needed training and mentorship to pathologists and can help in infrastructure development by adopting and validating new immunohistochemical stains. Persistent unresolved cases indicate that molecular techniques are indispensable in for diagnosis in a minority of cases. The development of affordable alternative molecular techniques may help with these histologically unresolved cases.

## Introduction

The World Health Organization (WHO), classification of central nervous system (CNS) tumors has traditionally relied on the morphologic appearance of the tumor under the light microscope. In recent years, however, molecular information has increasingly been used for diagnostic classification. The 4^th^ revised edition of the WHO released in 2016 introduced the concept of “layered integrated diagnosis,” according to which reporting of molecular alterations was made a formal part of the essential diagnostic criteria of several tumor entities (1, 2). This conceptual leap in diagnosing CNS tumors was made possible by the wide availability of next-generational sequencing (NGS) technologies. This led to the adoption of genetic sequencing as a routine clinical test in most academic centers in North America and Europe. This trend reached its zenith with the introduction of the 5^th^ edition of WHO classification in 2021 (CNS5), in which several tumor entities are now defined by their genomic or epigenomic signatures (3, 4). Several studies demonstrate that a multiomic approach improves diagnostic precision (5).

Molecular diagnostic techniques such as NGS and DNA-methylation profiling remain out of reach for most LMICs. Pathologists in the LMIC are either insufficiently aware of the recent diagnostic guidelines or their implementation remains outside their practical experience. As a result, there is a widening gap in diagnostic and patient management practices between LMICs and high-income countries (HICs). CNS5 criteria allow for the use of the suffixes “Not Otherwise Specified (NOS)” for cases in which the necessary diagnostic molecular tools are not available; however, this can result in many cases being assigned to these waste-basket categories (embryonal tumor, NOS; infiltrating glioma, NOS, and so on). The WHO classification purports to providing a shared vocabulary for communication and practice guidelines to pathologists worldwide. However, the utility and relevance of the CNS5 in LMIC remain to be demonstrated.

Various approaches have been implemented to enhance the diagnostic capacity in LMICs and to bring pathologists in these regions up to date with current diagnostic practices. One such approach is Institutional twinning, which refers to the collaboration and sharing of expertise and resources between institutions in LMICs and HICs. Here, we describe the impact of a 7-year-long twinning experience between Aga Khan University Hospital (AKUH) in Karachi, Pakistan, and the Hospital for Sick Children (SickKids) in Toronto, Canada, on histopathologic diagnosis. The twinning program had two components: a multidisciplinary tumor board (MDTB) meeting between AKUH and SickKids and a pathological review of biopsy material of selected cases at SickKids. Previously, we demonstrated the impact of twinning on neurooncological services (6). The results of the histopathological review of biopsy material at SickKids are described in more detail in this paper.

## Methods

The pediatric neuro-oncology twinning program between the Hospital for Sick Children (SickKids) in Toronto, Canada, and several hospitals in Pakistan began in June 2014. Pakistani partners included the Aga Khan University Hospital (AKUH) and Indus Children’s Cancer Hospital (ICCH) in Karachi as significant partners. The partnership was later expanded to include several private and public sector hospitals. Tissue biopsy and initial histopathologic processing were conducted locally at each hospital, but all pathology was later reviewed at the AKUH. At AKUH, the pathology department does not follow a subspecialty practice model for pathology. This means that any of the approximately 25 histopathologists available can review a CNS tumor case. However, most cases are reviewed by KM at some point. It is important to note that subspeciality fellowship training in neuropathology is currently unavailable in Pakistan.

A total of 460 cases were reviewed and discussed in the virtual (video-conferenced) multidisciplinary tumor board (MDTB) meetings during the study period (2014–2020). Typically, the meetings were arranged once every month and were attended by specialists from both countries. The Pakistani side was represented by neuro-oncologists, neurosurgeons, radiation oncologists, neuroradiologists, and neuropathologists, whereas one or more neurooncologists and neuropathologists represented SickKids in these meetings. The pathologists in Pakistan shared photomicrographs of *H&E* and immunohistochemical stains for each case. The case was then discussed and recommendations given for further treatment or pathology review. Select cases (n = 141 included in this study) were sent to SickKids for review. Inclusion criteria for such cases included the following: (1) cases in which a precise histopathologic diagnosis was not reached at AKUH; (2) cases requiring demonstration/ruling out of specific molecular alterations such as *IDH1/2*, histone 3 genes, and *BRAF* mutations; (3) unusual cases that required expert review for confirmation of the AKUH diagnosis; (4) any case for which the treating clinician requested a consult; and (5) consult was requested by the team during MDTB meetings. Patients who were 19 years old or younger at the time of biopsy were considered pediatric and included in this study. Typically, one to two blocks were sent for review and additional testing. Specimen shipping times typically varied from 7 to 10 calendar days, and preliminary diagnosis was typically rendered within 5–7 calendar days of receipt by the consulted pathologist.

Histologic processing conducted at the referring institutions followed standard guidelines in compliance with those of the College of American Pathologists (CAP). At the initiation of the study, both AKUH and Indus Hospital labs were in the process of acquiring CAP accreditation, receiving CAP accreditation in 2018 and 2023 respectively. Formalin-fixed paraffin-embedded (FFPE) blocks or unstained sections on glass slides, along with clinical information and official histopathologic reports, were sent along for review to SickKids. These cases were logged into the SickKids system and treated like any other referral case. H&E examination, immunohistochemical staining, and any relevant molecular test were then conducted at the discretion of the consulting neuropathologist (CH).

Molecular testing included fluorescent *in situ* hybridization (FISH), NanoString (7), or a TruSight Assay. Methodological details of the NanoString assay have been published before (7, 8). Briefly, custom panels (pediatric low-grade glioma panel, medulloblastoma panel, or the ependymoma fusion panel) were developed and tested using NanoString nCounter system (NanoString Technologies, Seattle, WA). RNA was extracted using the RNeasy FFPE kit ((QIAGEN, Valencia, CA). Probes designed to detect expression of three different housekeeping genes were included to assess RNA quality. For the medulloblastoma panel, probes were designed to detect gene transcripts enriched in specific groups including the following: WNT signature genes: *WIFI, TNC, GADI, DKK2*, and *EMX2; SHH* signature genes: *PDLIM3, EYAI, HHIP, ATOHI*, and *SFRPI*; Group 3 signature genes: *IMPG2, GABRA5, EGFL11, NRL, MAB21L2*, and *NPR3*; Group 4 signature genes: *KCNA1, EOMES, KHDRBS2, RBM24, UNCSD*, and *OASI*. Oligonucleotide probes were obtained from Integrated DNA Technologies (Coralville, IA), and the Elements tag sets were supplied by NanoString Technologies (Seattle, WA). A PAM class prediction algorithm was used to predict subgroup based on the expression levels of the above signature genes. The subgrouping was subsequently confirmed by visually inspecting the expression levels of the 22 signature genes. Pediatric LGG fusion gene analysis used probes designed to detect fusion transcripts in several genes most notably *BRAF*, *FGFR1*, and *FGFR3* genes. Similarly, the ependymoma fusion gene detection probes were designed to detect fusion transcripts including *C11orf95-RELA* and *YAP1-MAMLD1*.

Later in the course of the study, cases were tested using the TruSight pan Cancer RNA panel (Illumina, San Diego, CA) using FFPE tissue. NGS and Automated Fusion Calling RNA-derived NGS libraries are enriched using the TruSight Pan-Cancer 1385 gene panel. The TruSight Pan-Cancer-targeted gene list can be found at https://www.illumina.com/content/darn/illuminamarketing/documents/products/genelists/genelistTruSight pan cancer.xlsx). Libraries were sequenced on an Illumina MiSeqDx, with a minimum library size of two million reads. Sequence was aligned to the hg19 human genomic scaffold, and fusions are called using the Illumina STAR aligner (v2.5.0b) and the Manta structural variant caller (v1.5.0). The following genes were manually checked for fusions using IGV: *FGFR1, FGFR2, FGFR3, BRAF, RAF1, NTRK1, NTRK2, NTRK3.*

MMR testing by immunohistochemistry was performed in a subset of cases based on clinical suspicion or histomorphological features. MSH2, MSH6, MLH1, and PMS antibodies were used.

Since the study was conducted prior to the release of the 2021 World Health Organization (WHO) classification system, the official diagnoses used the WHO 2016 nomenclature.

For this study, each case was described as *concordant, subtyped, discordant*, or *deferred*. Cases were descried as discordant when there was a significant change in diagnosis often involving a change of grade, tumor cell lineage, etc. In subtyped cases, there was no change in diagnosis, but a tumor subtype was provided by the consulted pathologist. Cases in this category most commonly included glioblastoma and medulloblastoma. In concordant cases, there was either no change in diagnosis or a more specific diagnosis was provided without a change in the diagnostic class, for example when the expert diagnosis was a diffuse astrocytoma, IDH mutant instead of a referring diagnosis of diffuse glioma. In two cases, only a descriptive diagnosis was rendered by the expert neuropathologist.

## Results

The referring and consulted pathologists were in general agreement regarding the diagnosis in 102 cases (72.3%). Expert consultation provided subtyping without a change of diagnosis in 53 cases (*subtyped* cases). The rest, described here as *concordant* cases, often showed refinement of the diagnosis upon expert review.

**Figure 1:**
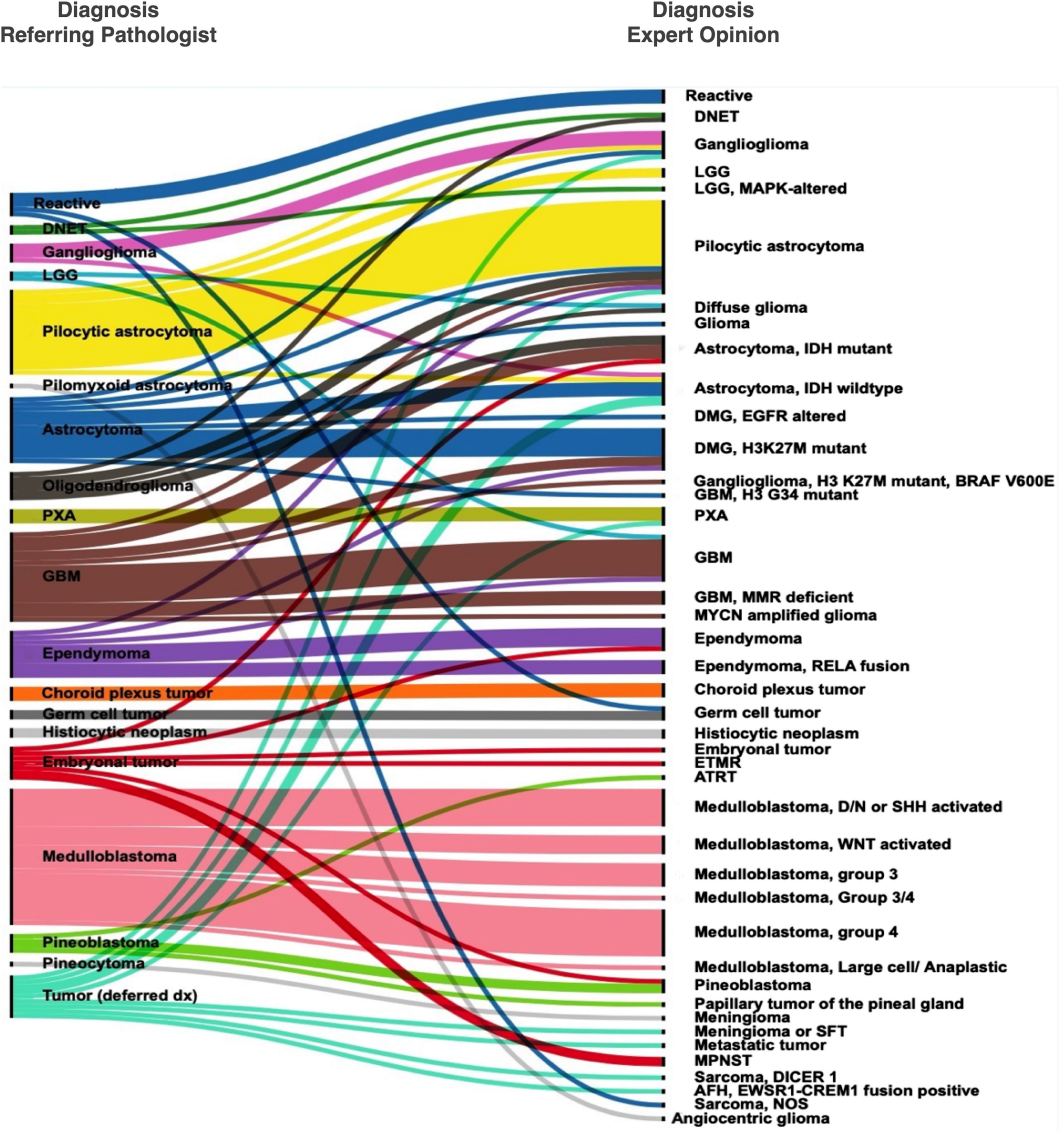
Concordance and discordance in histopathological diagnosis between the referring pathologists and the expert opinion.

Good concordance was seen for tumors, such as pleomorphic xanthoastrocytoma (PXA), choroid plexus tumors, pineoblastoma, and medulloblastoma. In these tumors, the expert opinion provided confirmation of the diagnosis and identification of molecular alterations. In 23/49 concordant cases, the consult identified or ruled out common driver genetic alterations in the diagnosed tumor type (**Table 1**, **Supplementary Table 1**). Of the 32 patients that were diagnosed with diffuse glioma, i.e., astrocytoma, anaplastic astrocytoma, glioblastoma, or high-grade glioma (HGG), histone alterations were found in a little over a third (nine cases of H3K27M, one case with *EGFR* ex 20 mutations, and one case of *H3 G34R* mutation), and IDH1 mutations in six (four with *IDH1 R132H* and two with *IDH1 R132S* mutations (**Table 1**, **Figure 1**).

**Table 1:** Molecular alterations identified upon consultation at SickKids.

Molecular alteration	Method of detection	Number of positive cases
***BRAF V600E***	IHC	**13**
***BRAF* fusion (KIAA1549 - BRAF)**	**NanoString assay**	**10**
*BRAF (ex16 - ex9)*		6
*BRAF (ex15 - ex9)*		3
*BRAF (ex16 - ex11)*		1
*BRAF* duplication	FISH	1
**Histone mutations**	**IHC**	**10**
*H3 K27M*		9
*H3 G34R*		1
***IDH* mutations**		6
*IDH1 R132H*	IHC	4
*IDH1 R132S*	NanoString	2
**Mismatch repair deficiency**	**IHC**	**5**
***c11orf95-RELA* fusion**	**NanoString assay**	**3**
***MYB-QKI* fusion**	**NanoString assay**	**1**
***MYCN* amplification**	**TruSight assay**	**1**
***KRAS* p.Q61K mutation**	**TruSight assay**	**1**
**FGFR3 mutation**	**TruSight assay**	**1**
***EWSR1-CREM* fusion**	**TruSight assay**	**1**
***EGFR* mutation**	**TruSight assay**	**1**
***DICER 1* mutations**	**TruSight assay**	**1**
***NF1* mutations**	**TruSight assay**	**1**
***SMARCB1* loss**	**IHC**	**1**
**Total**		**57**

**Figure 2:**
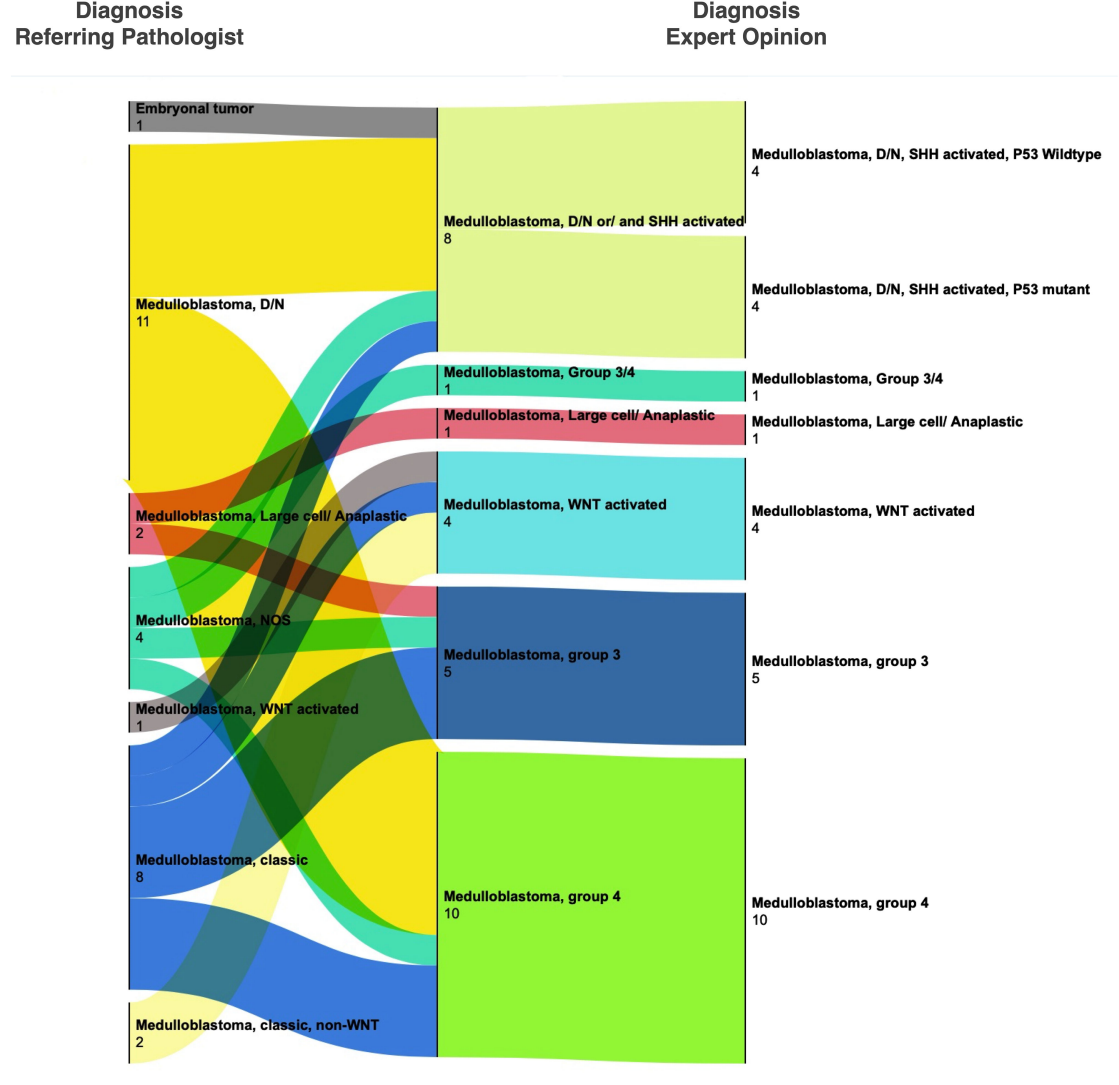
Change in histopathological diagnoses after expert consultation for medulloblastoma. Most cases were given a histologic diagnosis by the referring pathologist. Upon review at SickKids, cases received either molecular testing alone (by NanoString assay) or both molecular testing and histologic review. Significant discordance was seen in the interpretation of B-catenin immunohistochemistry and desmoplastic nodular histology (also see **Supplementary Table 1**).

Of the 29 medulloblastoma, 28 had been called accurately by the referring pathologists; in one case, the diagnosis was deferred. Expert consultation, however, provided molecular subclassification in these cases using an assay based on the NanoString nCounter system (8). In most cases of medulloblastoma, the consulted pathologist conducted histologic review in addition to molecular testing, providing an opportunity for the referring pathologist to compare their histologic diagnosis for WNT-activated subtype and desmoplastic/nodular subtype for which the immunohistochemical (B-catenin) or special histologic (reticulin) stains were available at AKUH. The results (**Figure 2**) show variable degrees of concordance for histologic subtypes of medulloblastoma. Discordant cases were due to differences in interpreting reticulin stain, not performing reticulin stain, and not recognizing patchy and often rare B-catenin nuclear positivity. GAB1 immunostain was validated at AKUH at the conclusion of the study and is now routinely performed to enable identification of SHH-activated subtype of medulloblastoma.

Seven cases were deferred to expert opinion or were diagnosed descriptively as malignant neoplasms, high-grade gliomas, or embryonal tumors. These eventually yielded a variety of low and high-grade tumors including an angiomatoid fibrous histiocytoma with *EWSR1::CREM* fusion, a pleomorphic xanthoastrocytoma, and a DICER 1 associated sarcoma.

A disagreement in diagnosis was seen in 30 cases (referred here as *discordant* cases). Discordant cases included changes in diagnoses with limited clinical impact such as a change from a Pilocytic astrocytoma to ganglioglioma and vice versa; as well as cases with major clinical impact such as change in tumor grade (high-grade to low-grade or vice versa), change from neoplastic to non-neoplastic or vice versa, or change of tumor type/lineage (such as a switch between embryonal, ependymal, glial categories). Notable cases including two cases which were deemed non-neoplastic were diagnosed as a germinoma, and a sarcoma (**Supplementary Table 1**). Both these specimens featured heavy inflammatory reaction, demonstrating the difficulty of accurately diagnosing cases where rare neoplastic cells are present alongside a majority of reactive or normal cells.

Seven out of 10 patients who were initially diagnosed with ependymal tumors at AKUH were called as such on expert consultation (**Figure 1** and **Supplementary Table 1**). The remaining three were astrocytomas (a GBM, a glioma with an *H3 K27M* mutation, and a pilocytic astrocytoma). Conversely, one patient diagnosed with a posterior fossa “CNS Embryonal tumor with rhabdoid features” was eventually diagnosed with ependymoma by the expert. These discrepancies could be avoided by recognizing the histologic features and immunohistochemical profiles of particular CNS-tumor types. To enable astrocytoma-ependymoma differentiation, Olig2 was validated and incorporated in the immunohistochemical repertoire at AKUH. Subtyping was provided in four ependymoma patients with identification of *RELA* fusion in three supratentorial ependymoma cases and posterior fossa-A designation in a fourth ventricular tumor.

Not unexpectedly, poorly differentiated tumors diagnosed as embryonal tumors often changed diagnosis upon expert review with the final diagnosis being a glioblastoma with *IDH1* mutation, an ependymoma (mentioned above), and a peripheral nerve sheath tumor. These cases show the inability of histologic examination to distinguish between ependymoma, astrocytoma, and embryonal tumors in poorly differentiated, highly malignant cases.

Another tumor type with a high degree of discordance in this series is oligodendroglioma. Six specimens diagnosed as oligodendroglioma or likely oligodendroglioma were eventually diagnosed as (*IDH1* mutant astrocytoma, two cases; one DNET and two pilocytic astrocytoma and a diffuse glioma). Two cases showed a major change in grade between low-grade and high-grade, whereas two additional cases showed a change between diffuse vs. circumscribed glioma.

Apart from providing confirmation, refining diagnoses, and correcting some diagnoses, institutional twinning helped with diagnostic capacity building at AKUH. Several new immunohistochemical stains to aid with the diagnosis of CNS tumors were validated at AKUH and incorporated in the clinical laboratory’s testing menu. For this purpose, SickKids shared protocols and cases already tested at SickKids were used for validation. Newly introduced stains included stains used for differentiation between cell lineages (Olig2), surrogate markers for molecular alterations (IDH1 R132H, ATRX), markers of specific tumor types (Lin28 for ETMR and L1CAM for supratentorial ependymoma, RELA/ZFTA fusion-positive), and markers for tumor subtyping (including GAB1 for SHH-activated tumor and H3 K27me3 for ependymoma, posterior fossa-A/posterior fossa-B distinction). Introducing these stains developed in-house capacity to resolve additional cases, thus reducing dependency on expert review at SickKids for such cases toward the conclusion of the study. IDH1 R132H stain was incorporated in 2019. In the beginning, a few cases showed differing interpretation of this stain between AKUH and SickKids Pathologist; since then, there has been good concordance. Immunohistochemical stains for identifying mismatch repair deficiency (MMR) were introduced at AKUH in 2019 and were performed in a few cases in this cohort with concordant results on retesting at SickKids Hospital. Additional immunostains are in the validation process (including immunostains for *H3 K27M*, *H3 G34 R/V*, and *BRAF V600E*).

## Discussion

We describe the impact of expert opinion and mentorship provided during institutional twinning on histopathology diagnosis. We also show the types of unresolved cases and those with the most discordant diagnosis between the referring LMIC pathologists and HIC expert opinion.

The relevance of molecular diagnostics-based criteria like the CNS5 for LMICs has been called into question, and it has been suggested that, increasingly, these criteria are unlikely to be of significant benefit to most patients in LMICs (9). Several authors have pointed out that precision therapies are still largely out of reach of most patients in LMICs. Hence, the identification of molecular biomarkers and diagnostic criteria heavily based on molecular alterations is largely irrelevant to LMICs.

Molecular testing and identification of oncogenic drivers and prognostic and therapeutic markers have also intensified the search for surrogate markers that can be obtained using traditional diagnostic techniques. Surrogate biomarkers for molecular alterations in central nervous system (CNS) tumors are less expensive than other forms of molecular testing. Still, they are also faster and easily integrated into the usual surgical pathology workflow. These biomarkers can be diagnostic, prognostic, and/or predictive (e.g., provide biological targets for treatment). We also note that in rare instances, successful targeted therapies have been conducted in LMICs, including by our group (10, 11).

We show that most cases will likely be resolved by careful histopathologic analysis and the use of immunohistochemical stains, including surrogate stains for histone 3, *IDH1* and *BRAF* mutations, and subtype-specific stains for ependymoma and medulloblastoma. Out of a total of 57 genetic alterations identified in the cases in this study, only 10 cannot be identified by IHC stains. Half of all medulloblastoma consisted of WNT-activated or SHH-activated groups, which can be diagnosed based on immunohistochemical stains. The remaining unresolved cases (10 cases with non-*BRAF*/non *RELA/ZFTA* fusions and 16 non-WNT/non-SHH medulloblastoma) can then be subjected to NanoString, NGS, or other advanced molecular testing methods.

As shown in this and previous studies, *BRAF-KIAA1549* fusions and mutations such as *BRAF V600E*, *IDH1 R132H*, and *H3 K27M* are among the most common types of genetic alteration mutations in pediatric gliomas, and the immunostains for these alterations should be part of the immunohistochemistry (IHC) repertoire of reference labs in LMICs. These stains should be used in combination with Olig2 (to identify H3 G34R/V mutant tumors), ATRX, and P53 stains. Therefore, in many cases, the likely diagnosis can be achieved by using surrogate immunohistochemical markers in the context of the clinical features. This approach will miss a small minority of IDH mutant tumors, namely, those with non-canonical *IDH* mutations (enriched in specific clinical scenarios such as the infratentorial diffuse gliomas (12) and Li-Fraumeni patients) (13).

Although morphological features alone can be used for diagnosis of CNS tumors in a vast majority of cases; in reality, pathologists often use the identification of molecular biomarkers to substantiate their histologic impression. For example, the identification of a *BRAF* fusion can lend credence to a diagnosis of histologically ambiguous pilocytic astrocytoma, or the presence of *BRAF V600E* mutation and deletion of *CDKN2A/B* gene by FISH or DNA testing can confirm the diagnosis of PXA. Similarly, although not strictly required, identification of *MYB-QKI* fusion can confirm a diagnosis of angiocentric glioma, which can be confused with other LGGs such as pilomyxoid astrocytoma, as evident in this series. Limited access to molecular testing places additional demands on the clinical and diagnostic acumen of both pathologists and oncologists who ought to recognize each tumor’s standard, expected behavior, and treatment response, so that if a particular patient deviates from that pattern, advanced, more costly diagnostic tests are obtained to rule out alternative diagnoses.

Our data show that in a significant subset of cases, the correct diagnosis could have been arrived at by careful study of the patient’s clinical picture, astute histologic examination, and greater awareness of the published diagnostic criteria. This is exemplified by the diagnosis of oligodendroglioma in six patients; none of them was eventually substantiated as an oligodendroglioma. According to 2016 and 2022 WHO diagnostic criteria, this diagnosis should only be given to tumors that are IDH mutant and 1p/19q co-deleted. Furthermore, oligodendroglioma will be exceedingly uncommon in the pediatric age group. Knowledge and expertise gaps were therefore at least partially responsible for this discrepancy. We expect that this issue will be partly resolved by subspecialty-based practice by virtue of which a pathologist specializes in providing CNS tumor diagnosis either after a structured fellowship training or by learning on the job. IDH1 R132H immunohistochemical and ATRX stains are now available at AKUH and at least two other laboratories in the country and will hopefully facilitate the diagnosis of oligodendroglioma. 1p/19q co-deletion testing, the other requirement for oligodendroglioma diagnosis, is currently available in only two laboratories in Pakistan, namely, AKUH and Shaukat Khanum Memorial Cancer Hospital (SKMH), Lahore. The cost of this fluorescent *in situ* hybridization (FISH)-based test is borne out of pocket by the family/patient and, at approximately 100$, is often considered prohibitive unless strongly advised by the treating physician.

In a minority of cases, advanced molecular testing for identification of the characteristic molecular alteration was required for the diagnosis. This includes a case of angiomatoid fibrous histiocytoma with *EWSR1-CREM* fusion, an angiocentric glioma with *MYB-QKI* fusion, a glioma with *MYCN* amplification, two patients with non-canonical IDH mutations (both with *IDH1 R132S*), and a patient with a *KRAS* mutation (**Table 1**).

At the beginning of the study, AKUH did not provide any molecular testing for the diagnosis of CNS tumors. In recent years, testing for *IDH1/2* hotspot mutations has been incorporated, but gene fusion testing, mutation testing for *BRAF* or histone genes, and copy number testing for *CDKN2A* deletion are still not available. We also note that the diagnostic criteria for CNS tumors have undergone significant revision since the conclusion of this study. The WHO 2021 classification of CNS tumors has increased the utility of NGS and DNA-methylation assays in the diagnosis of CNS to the extent that a significant number of tumors, particularly gliomas, cannot be classified on histology or IHC alone.

We believe that subspecialty practice for neuropathology, at least in a handful of reference labs in a particular country or region, will improve histopathologic diagnosis. This mirrors our experience that developing a subspecialty caregiver team in which the caregivers become experts in their respective fields improves patient outcomes for CNS tumors (6). Pediatric neuropathology is complex by its very nature. Tumors are histologically and molecularly diverse. The field is rapidly growing with frequent advances and changes to diagnostic criteria. In addition, the incidence of these tumors is low; hence, a general pathologist will see only a small number of cases in a certain month or year. It, therefore, stands to reason that a general pathologist cannot be expected to master the intricacies of this field. Sub-specialization is needed. The case volumes in many reference laboratories (such as AKUH and SKMH) can sustain this model. Such sub-specialization has already taken place in other aspects of pediatric neuro-oncology care where specialized pediatric neuro-oncologists, pediatric neurosurgeons, and often pediatric neuroradiologists now care for cases of CNS tumors in children. Studies have shown improved patient outcomes due to the development of subspecialty caregiver teams in which the caregivers become experts at their respective fields (6).

We previously showed that discordance in clinical plans between AKUH and SickKids decreased from around 30% at the beginning of the twinning to 16% at the end of the 7-year study period (2014–2020, both inclusive) (6). In contrast, the number of cases with discordant diagnoses remained high throughout the study period, perhaps reflecting the role of molecular testing in reaching an integrated diagnosis (6). Stated another way, whereas additional training and subspeciality focus will solve some of the problems, they are unlikely to improve the discrepancies further, as even the most experienced neuropathologist will render a somewhat descriptive diagnosis without molecular results.

Our study supports the findings of several previous studies showing the role of second review in improving diagnostic accuracy. A retrospective review of pediatric tumor cases received at St. Jude Children’s Research Hospital (SJCRH) from international institutions showed major disagreement in approximately 25% of cases overall and 33% in the CNS (14). The rate of major disagreement at US institutions was lower than that for international institutions at. A switch from malignant to less aggressive (GBM to PXA, for example) was three times more common than vice versa. This study, which compiled data from 2009 to 2011, identified lack of the availability of immunohistochemistry as a major cause for the discrepancy (14). Whether the problem of inadequate tools leading to diagnostic inaccuracy has further aggravated in the molecular era remains to be seen. Another major cause identified by the study was deficient training of pathologists in the diagnosis of pediatric neoplasms. Another study by the same investigators focused on training of a general pathologist in the diagnosis of pediatric neoplasms, implementation of a basic IHC panel in a pathology laboratory in a developing country, and inclusion of the pathologist in a multidisciplinary team. These measures dramatically improved the diagnostic accuracy of pediatric neoplasms (14). This group showed that brief, focused training in pediatric cancer histopathology improved diagnostic accuracy (15). Similarly, a study from Lebanon identified the unavailability of immune and molecular stains as the primary cause of diagnostic discrepancy, accounting for 12/14 cases. The remaining two were due to differences in interpretation (16)..

We demonstrate the utility of remote/virtual twinning between an LMIC and an HIC. While most twinning programs involve physical exchanges of personnel between the participating institutions—a time-consuming and costly proposition—we show the feasibility of virtual twinning in combination with the mailing of pathology specimens. Similar results were shown by Qaddoumi and colleagues achieving successful outcomes using telemedicine-based twinning between King Hussein Cancer Center, Amman, Jordan, and SickKids (17). Interestingly and of particular relevance to this discussion, the most common recommendation was a review of the neuropathology, which was suggested in 10/23 patients. This resulted in a change in the initial diagnosis or the grading of the tumor with significant consequences in terms of subsequent management. As a result, six patients were recommended observation instead of radiation, thereby saving resources and long-term treatment-related toxicity for those patients (17). In a follow-up paper in 2018, the authors presented a 10-year review of their experience (18). These authors noted that during the study period, there were suggestions for molecular testing, including BRAF fusion/mutation, medulloblastoma subgrouping, and genetic testing. Six cases underwent such testing (18). In one case of disseminated recurrence of a pleomorphic xanthoastrocytoma, identifying *BRAF* mutation at the SickKids laboratory led to the administration of BRAF inhibitor therapy (11).

Recently, important initiatives have been launched to improve access to high-quality medicines and technologies in LMIC by strengthening training programs and developing centers of excellence. One such initiative is the World Health Organization’s Global Initiative for Childhood Cancer. Established in 2018, this initiative brings together stakeholders from around the world with the joint goal of increasing the survival rate of children with cancer globally to at least 60% by 2030 while reducing their suffering and improving their quality of life (19). We hope that histopathologic and molecular diagnostics will not be neglected in this and other similar initiatives. We also note that this study was concluded in 2020 before the widespread adoption of DNA methylation-based classification for diagnosing challenging cases. None of the cases in this cohort were tested on that assay. It is conceivable that some of the cases unresolved by traditional histologic and immunohistochemical stains and NGS studies will be resolved using DNA methylation array-based testing. Similarly, several cases were diagnosed as glioblastoma, which is no longer a favored term in the pediatric and young adult age groups. In short, in 2024 as compared with the study period, the diagnostic requirements have become even more complicated and resource intensive.

While this paper only describes in detail the neuropathology infrastructure at AKUH, Karachi, we note that other leading laboratories in Pakistan face similar limitations. A large chunk of all CNS tumors in Pakistan are eventually reviewed at a handful of laboratories in the three major metropolitan cities in Pakistan. These laboratories include AKUH in Karachi, Shaukat Khanum Memorial Cancer Hospital (SKMH) and Chughtai Lab in Lahore, and Shifa International Hospital in Islamabad. AKUH currently offers the most extensive immunohistochemical panel of these institutions. SKMH has recently validated an NGS-based DNA mutation panel, hopefully leading to better identification of key diagnostic, therapeutic, and prognostic markers for CNS tumors in Pakistan. A fusion panel is currently not being offered at any institution in Pakistan.

One possible limitation of LMIC-HIC twinning programs is that it may result in overreliance on second opinion. Pathologists in LMIC should diagnose cases as best as possible based on available tools rather than relying solely on HIC experts or molecular tests. Twinning between LMIC and HIC institutions is maximally beneficial when aiming to build capacity in LMIC. A second opinion from an HIC expert cannot replace local experts.

In conclusion, this study identifies persistent gaps in diagnosing CNS tumors in LMICs due to unavailability of specialized immunohistochemical stains, molecular diagnostic tools, and deficiencies in pathologists skill and knowledge. Twinning between LMIC and HIC institutions can mitigate these deficiencies, help in capacity building, and, therefore, greatly benefit patients. Previously, we showed the role of twinning in improving the care of patients with pediatric CNS tumors in Jordan and Pakistan (6, 16). We now show its impact on histopathologic diagnosis.

## Data availability statement

The original contributions presented in the study are included in the article/**Supplementary Material**. Further inquiries can be directed to the corresponding author.

## Ethics statement

The studies involving humans were approved by The Aga Khan University - Ethics Review Committee. The studies were conducted in accordance with the local legislation and institutional requirements. Written informed consent for participation in this study was provided by the participants’ legal guardians/next of kin.

## Author contributions

AG: Conceptualization, Data curation, Formal analysis, Investigation, Writing – original draft, Writing – review & editing. NM: Data curation, Funding acquisition, Investigation, Methodology, Project administration, Writing – review & editing. MS: Formal analysis, Writing – review & editing. AA: Writing – review & editing, Data curation, Formal analysis. ZS: Writing – review & editing, Data curation, Formal analysis, Visualization. EB: Conceptualization, Funding acquisition, Methodology, Resources, Supervision, Writing – original draft, Writing – review & editing. UT: Conceptualization, Investigation, Methodology, Resources, Supervision, Writing – review & editing. CH: Conceptualization, Data curation, Formal analysis, Funding acquisition, Investigation, Methodology, Resources, Supervision, Validation, Writing – review & editing. KM: Conceptualization, Data curation, Formal analysis, Funding acquisition, Investigation, Methodology, Resources, Writing – original draft, Writing – review & editing.

## Supplementary material

Supplementary Table 1 Clinicopathologic information and summary of diagnosis rendered by the referring pathologist and the expert neuropathologist at SickKids.
